# Cleavage/Alteration of Interleukin-8 by Matrix Metalloproteinase-9 in the Female Lower Genital Tract

**DOI:** 10.1371/journal.pone.0116911

**Published:** 2015-01-22

**Authors:** M. Reza Zariffard, Kathryn Anastos, Audrey L. French, Elisaphane Munyazesa, Mardge Cohen, Alan L. Landay, Gregory T. Spear

**Affiliations:** 1 Department of Immunology/Microbiology, Rush University Medical Center, Chicago, Illinois, United States of America; 2 Departments of Medicine and Epidemiology & Population Health, Albert Einstein College of Medicine and Montefiore Medical Center, Bronx, New York, United States of America; 3 Ruth M. Rothstein CORE Center, Stroger Hospital of Cook County, Chicago, Illinois, United States of America; 4 Rwanda Biomedical Center, National Reference Laboratory Division, Laboratory Quality Assurance Direction, Kigali, Rwanda; Midwestern University, UNITED STATES

## Abstract

**Objective:**

Interleukin-8 (IL-8, CXCL8) plays important roles in immune responses at mucosal sites including in the lower genital tract. Since several types of bacteria produce proteases that cleave IL-8 and many types of bacteria can be present in lower genital tract microbiota, we assessed genital fluids for IL-8 cleavage/alteration.

**Study Design:**

Genital fluids collected by lavage from 200 women (23 HIV-seronegative and 177 HIV-seropositive) were tested for IL-8 cleavage/alteration by ELISA.

**Results:**

IL-8 cleaving/altering activity was observed in fluids from both HIV-positive (28%) and HIV-negative women (35%). There was no clear relationship between the activity and the types of bacteria present in the lower genital tract as determined by high-throughput sequencing of the 16S rRNA gene. Protease inhibitors specific for matrix metalloproteinases (MMPs) reduced the activity and a multiplex assay that detects both inactive and active MMPs showed the presence of multiple MMPs, including MMP-1, -3, -7, -8, -9, -10 and -12 in genital secretions from many of the women. The IL-8-cleaving/altering activity significantly correlated with active MMP-9 as well as with cleavage of a substrate that is acted on by several active MMPs.

**Conclusions:**

These studies show that multiple MMPs are present in the genital tract of women and strongly suggest that MMP-9 in genital secretions can cleave IL-8 at this mucosal site. These studies suggest that MMP-mediated cleavage of IL-8 can modulate inflammatory responses in the lower genital tract.

## Introduction

The chemokine Interleukin-8 (IL-8, CXCL8) is a member of the CXC chemokine family that plays a number of important roles in immunity including activation and attraction of neutrophils [1,2]. In vitro, IL-8 is produced by neutrophils, macrophages, monocytes and epithelial cells when exposed to either microbial products derived from commensal bacteria or organisms that cause sexually transmitted infections (STI) [3–5]. IL-8 levels in lower genital tract secretions are increased in women with STIs [5–7] and also increased in response to non-STI alterations in lower genital tract microbiota [7–11]. IL-8 elevations in genital secretions and in cultures of epithelial cells have been used as a biomarker of inflammation in clinical trials of microbicides [12,13].

There are several proteases that have been reported to act on IL-8. A protease made by some strains of *Streptococcus pyogenes* has been shown to cleave the C-terminal alpha helix of IL-8 resulting in IL-8 inactivation [14]. In infected patients, disease severity correlated with the IL-8 protease activity expressed by the *S. pyogenes* isolates [15]. *Porphyromonas gingivalis*, an oral bacterium implicated in periodontitis, also produces a protease that can cleave at the amino terminal end of IL-8 [16,17]. The host-derived protease matrix metalloproteinase-9 (MMP-9) has also been reported to cleave IL-8 [18]. MMP-9 removes six amino acids from the N-terminal portion of IL-8, but instead of inactivation, this cleavage results in dramatically enhanced IL-8 signaling through CXCR1.

The bacterial microbiota in the female lower genital tract of different women can be comprised of many different genera of bacteria including *Lactobacillus, Prevotella, Megasphaera, Gardnerella, Mobliluncus, Atopobium, Parvimonas, and Sneathia* [19–21]. Strains of *Streptococcus* and *Porphyromonas* are also present in the genital tract of some women. We hypothesized that some of these genital bacteria could express proteases that cleave IL-8. To explore this hypothesis, we used ELISA to assess a reduction of IL-8 detection after incubation with genital tract fluids collected from 200 different women. Since a reduction of reactivity in the ELISA does not necessarily show cleavage of IL-8, we used the terms “cleavage/alteration” and “cleaving/altering” throughout the paper to indicate reduction in ELISA activity.

## Material and Methods

### Subjects

Genital samples were obtained from women in the Rwanda Women’s Inter-association Study and Assessment (RWISA). RWISA is an observational prospective cohort study investigating the effectiveness and toxicity of antiretroviral therapy (ART) and comorbidities in HIV-infected Rwandan women.

Written informed consent was obtained in accordance with protocols approved by the Rwanda National Ethics Committee and the Institutional Review Board of Montefiore Medical Center, Bronx NY. Genital samples were collected by cervicovaginal lavage (CVL) performed by irrigation of the cervix with 10 ml of nonbacteriostatic sterile saline, followed by aspiration from the posterior fornix. CVL were transported from the study site to the lab within two hours of collection, aliquoted and frozen.

### Measurement of IL-8 cleavage/alteration

IL-8 cleavage/alteration was measured similarly to previously reports [14]. CVL were clarified by centrifugation and diluted 1:4 with RPMI-1640 medium buffered with HEPES (Sigma, St. Louis, MO, added as a source of cations) and 0.1 ml of diluted fluid was added to 0.1 ml (2 ng/ml) of carrier-free recombinant human IL-8 (rhCXCL-8/IL-8, R&D Minneapolis, MN, USA). The mixtures were incubated for 20 h at 4°C or 37°C. Afterward, ELISA was used to determine the concentration of IL-8 (BD Bioscience, San Diego, CA USA). The cutoff for determining if samples were positive for IL-8 cleavage/alteration was set by calculating the standard deviation of negative controls in multiple runs and multiplying by 3. In some experiments, protease inhibitors were added to the incubations; either General Protease Inhibitor (1:25 final concentration) (Sigma), EDTA (2 mmol final concentration), Marimastat (13 nM final concentration, Tocris, Bristol, UK) or CP471474 (16 nM final concentration, Tocris). A culture supernatant from Group A *Streptococcus* was used as a positive control in all experiments (a kind gift from Paul Sumby, Center for Molecular and Translational Human Infectious Diseases Research, Huston, Texas 77030).

### Pyrosequencing of the 16S rRNA gene and identification of bacteria

Bacteria in CVL were pelleted by centrifugation and DNA was isolated using the Fast DNA Spin Kit for Soil (MP Biomedicals, Solon, OH USA). Multitag Pyrosequencing, as described previously [22,23], was performed using 12 bar-coded primer sets each containing the 27F and 355R 16S rRNA gene primers. The Bayesian Classifier provided by the Ribosomal Database II Project (RDP 10) using forward reads only was applied to identify bacteria.

### Assays for MMPs

Total MMPs in CVL (both active and latent forms) were measured by a multiplex Luminex assay (R&D Systems Inc. Minneapolis, MN). The plate was read on a Luminex analyzer (LuminexH-Bio-PlexH 200, BIO-Rad Hercules, CA ). The sensitivity of detection of the different MMPs was as follows; MMP-1, 1.1 pg/ml; MMP-7, 7.3 pg/ml; MMP-7, 6.6 pg/ml; MMP-8, 16.6 pg/ml; MMP-9, 13.7 pg/ml; MMP-10, 3.2 pg/ml; and MMP-12, 0.7 pg/ml. A fluorogenic substrate that was susceptible to cleavage by multiple MMPs was used to measure active MMPs in CVL (OmniMMP, Enzolifesciences, Farmingdale, NY). CVL was assayed at 1:5 dilutions with 20 μl of OmniMMP substrate (final concentration 10 uM). The plate was incubated at 37°C for 1 h, and analyzed with excitation of 328 nm and detection of 393 nm. To measure activated MMP-9, an immunocapture system was used (R&D Systems Inc. Minneapolis, MN). The minimum detectable dose (MDD) of active MMP-9 was 0.1 ng/ml.

### Statistical Analysis

Statistical analysis was performed using the Instat statistical software package (GraphPad Software). Nonparametric and parametric methods were used for comparisons and to assess correlations, as indicated in the text and figure legends. A p-value of 0.05 or lower was considered significant.

## Results

### Cleavage/alteration of Recombinant human IL-8 by genital mucosal fluid

**Figure 1:**
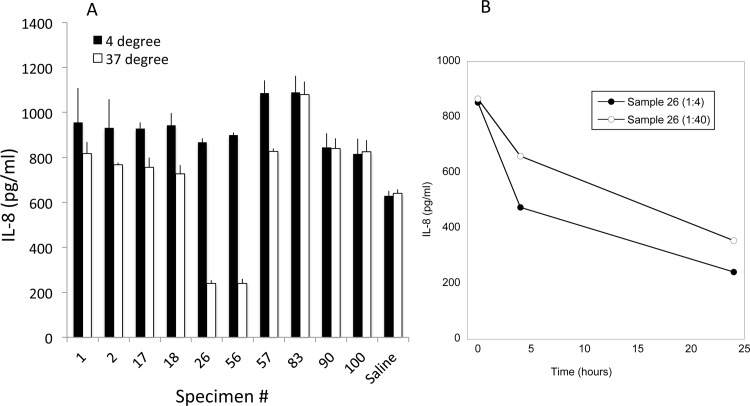
Cleavage of IL-8 in genital mucosal fluids. A. Recombinant IL-8 was added to either saline (negative control) or genital fluids (diluted 1:4) from 10 HIV-seropositive subjects and incubated at either 4°C or 37°C for 20 h. Levels of IL-8 were then measured by ELISA. The mean ± SD are shown. B. Recombinant IL-8 was added to genital fluid from subject 26 diluted 1:4 or 1:40 and incubated for 4 or 24 h before detection by ELISA.

An assay similar to one previously reported [14] was used to determine if proteases that could cleave IL-8 were present in the lower genital tract fluid collected from women. Recombinant human IL-8 was incubated for 20 h at either 4°C or 37°C with either saline (negative control), a supernatant from cultures of group A Streptococcus (positive control) or genital mucosal fluid collected by lavage from women. The positive control resulted in 95–100% reduction in ELISA detection (not shown) while incubation with the negative control had no effect on IL-8 (Fig. 1A). Samples from 200 women were tested; 23 from HIV-seronegative women and 177 from HIV-seropositive women. Reduction in IL-8 detection greater than 3% was observed in 57 (29%) of the 200 samples. Fig. 1A shows examples of several of the positive samples including ones from HIV+ subjects with the most reduction (subjects 26 and 56) and three from HIV+ subjects that exhibited no reduction (83, 90 and 100). Note that the genital fluid samples from the subjects had higher levels of IL-8 after 4°C incubation than the saline control because genital fluid from most women contains some endogenous IL-8 [7].

Cleavage/alteration was observed in 28% of the samples from the HIV-seropositive women and 35% from the HIV-seronegative women (p>0.05, Chi-square analysis). For the samples from the HIV+ women, we determined if there was a relationship between peripheral blood CD4 number (assessed by flow cytometry) and IL-8 cleaving/altering activity. Surprisingly, we found that the CD4 number was significantly higher in women whose samples were positive (>3% cleavage) for activity (median CD4 of 277 cells/microliter) than in those negative for IL-8 cleavage/alteration (<3% cleavage, median CD4 of 176 cells/microliter, p = 0.004, Mann-Whitney test). In contrast, the plasma HIV levels were not significantly different between the two groups.

The fact that IL-8 cleavage/alteration occurred at 37°C when compared with 4°C suggested that an enzyme was responsible. To determine the heat stability of the activity, a portion of one genital fluid sample was heated at 80°C for 45 min before testing its activity. While the unheated genital fluid reduced IL-8 detection by 55%, heating at 80°C completely eliminated the activity (0% cleavage) indicating the activity was heat labile (data not shown).

The time and concentration dependence of the IL-8 cleaving/altering activity in the sample from subject 26 was also tested. Substantial IL-8 cleavage/alteration was observed by 4 hours when the genital sample was diluted 1:40 with even more cleavage from the more concentrated sample (Fig. 1B). Incubation for 24 hours increased the amount of cleavage/alteration.

### Relationship between genital microbiota and IL-8 cleavage

Since previous studies had shown that IL-8 could be cleaved by proteases produced by the bacteria *Porphyromonas* and *Streptococcus* [14,16], we investigated a possible bacterial source for the activity. DNA was isolated from the bacterial pellets from nine genital fluid samples with activity and from three with no activity and the bacteria in the samples were identified by high-throughput sequencing of the V1/V2 region of the 16S rRNA gene.

**Figure 2:**
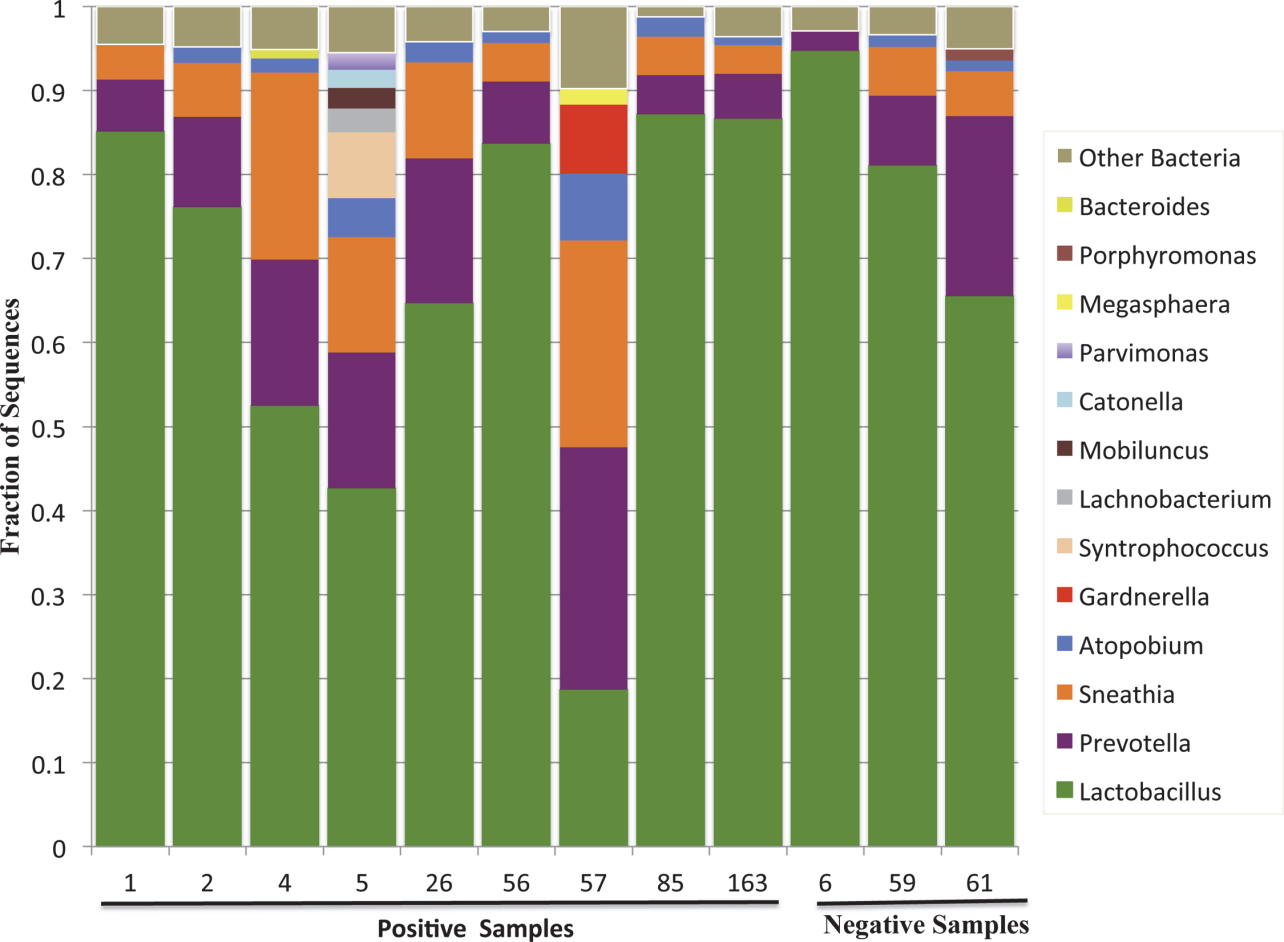
Lower genital tract microbiota in samples with or without IL-8-cleaving activity. The microbiota in the lower genital tract of women with or without IL-8-cleaving activity was identified by pyrosequencing of the 16S rRNA gene. The 13 most predominant bacterial genera are graphed.

Sequences corresponding to *Lactobacillus* were found in all samples and accounted for >50% of the sequences in 7/9 of the IL-8 cleavage/altering samples and 3/3 of the samples with no activity (Fig. 2). Sequences corresponding to *Prevotella* and *Sneathia* were detected in 12/12 samples and 11/12 samples respectively. Sequences corresponding to *Streptococcus* were not found in any of the samples. *Porphyromonas* sequences were found in one of the active samples (subject 5). A few genera, including *Gardnerella, Megasphaera* and *Syntrophococcus* were present only in samples positive for the activity, but were only found in only one or two of the positive samples. Thus, overall there was no bacterial genus that correlated closely with the IL-8 cleaving/altering activity and the bacterial profiles of the samples with the highest activity (26 and 56) were similar to the profiles of samples with no activity (Fig. 2).

### MMPs and IL-8 cleavage/alteration

**Figure 3:**
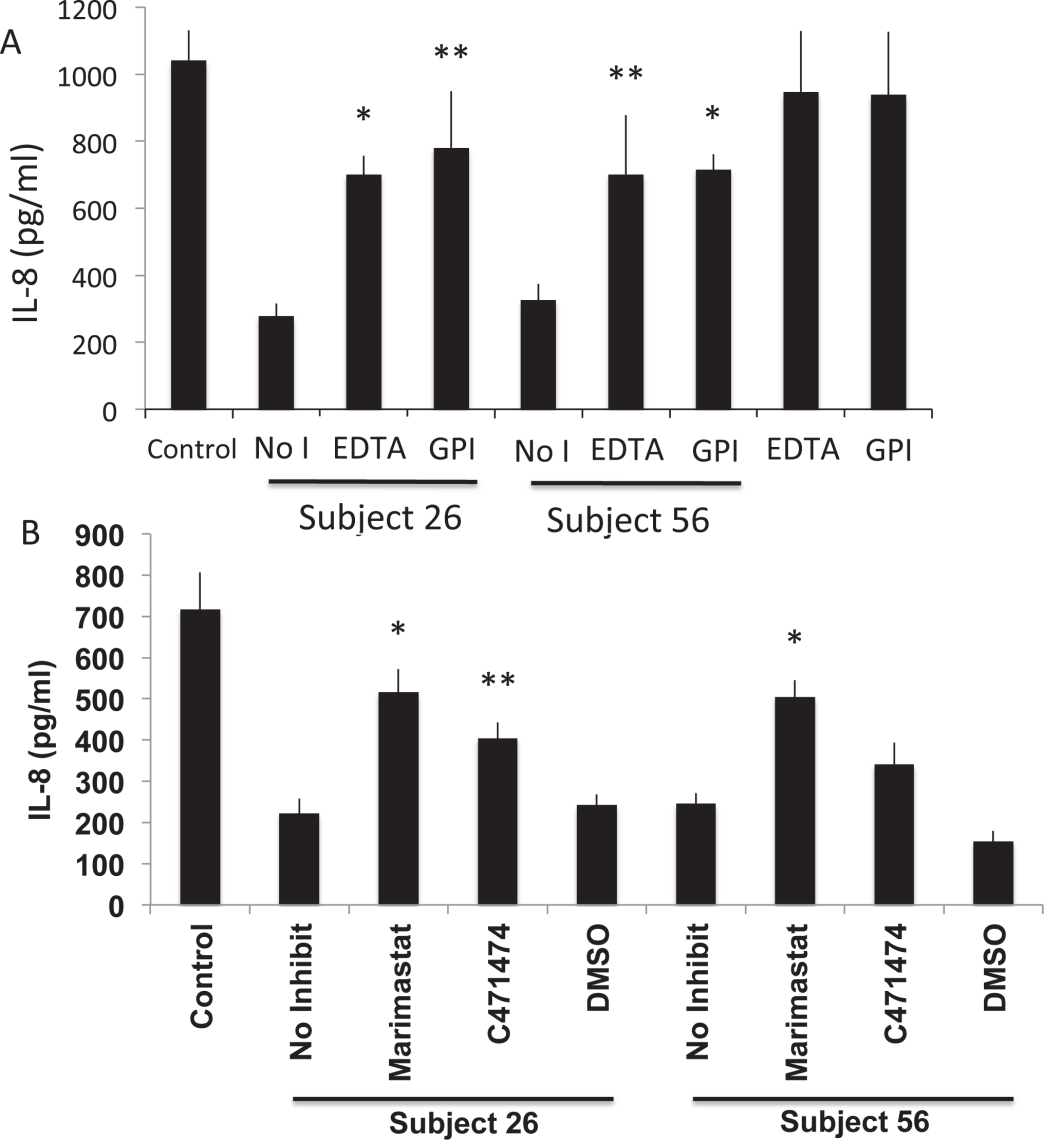
The effect of protease inhibitors on the IL-8-cleaving activity in genital tract fluid. A. Two samples positive for IL-8 cleavage (samples 26 and 56) were incubated with recombinant IL-8 for 20 h at 37°C in the presence or absence (No I) of EDTA or a cocktail of protease inhibitors (general protease inhibitor, GPI). B. MMP inhibitors Marimastat and C471474 were tested. For both A and B, control is IL-8 incubated without genital fluid or inhibitors. Samples were analyzed at 20 h by IL-8 ELISA. Shown are the mean values ± SD. * p<0.05, **p<0.1, t test compared to no inhibitor.

Since no clear relationship between the bacterial microbiota and IL-8 cleavage/alteration was observed, the type of protease responsible for the activity was next investigated. Samples with high activity (samples 26 and 56) were tested in the presence of several protease inhibitors. PMSF, a serine protease inhibitor, had no effect on activity (not shown). However, both EDTA and a protease inhibitor mixture (general protease inhibitor, GPI) reduced IL-8 cleavage/alteration with similar efficacy (Fig. 3A). Since EDTA is known to inhibit matrix metalloproteinases (MMPs), we next tested the effect of two MMP inhibitors, Marimastat and C471474, on IL-8 cleavage. Both of the MMP inhibitors reduced IL-8 cleavage/altering compared to the DMSO control, although Marimastat was more effective (Fig. 3B).

**Figure 4:**
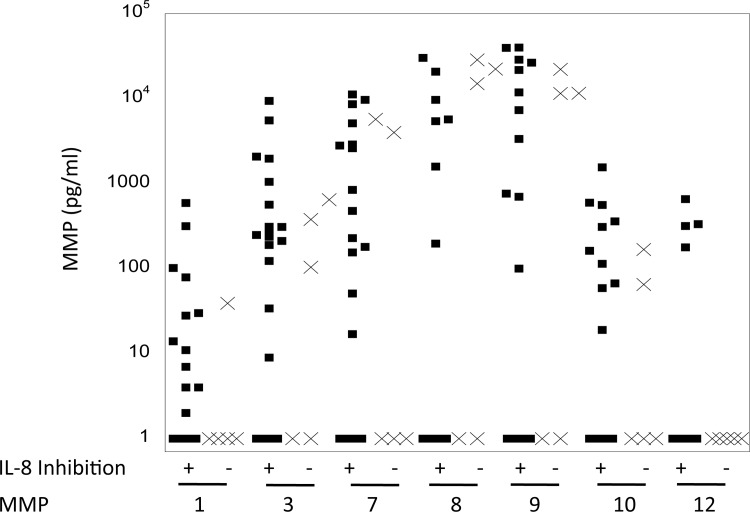
Total MMP levels in mucosal fluid samples. The concentration of MMPs 1, 3, 7, 8, 9, 10 and 12 were measured by Luminex immunoassay in mucosal fluid samples. 22 samples positive for IL-8 cleavage (square symbols) and 5 samples negative for IL-8 cleavage (x symbols) were evaluated.

Since the inhibitor experiments suggested MMPs could be involved in IL-8 cleavage/alteration, we next used a multiplex assay to measure the total amount (both active and inactive) of MMPs 1, 3, 7, 8, 9, 10 and 12 in samples that were positive (n = 22) or negative (n = 5) for activity (Fig. 4). All of the MMPs could be detected in at least some of the samples. When comparing IL-8-cleavaging/altering-positive and –negative samples, none of the MMPs were significantly different between the two groups.

**Figure 5:**
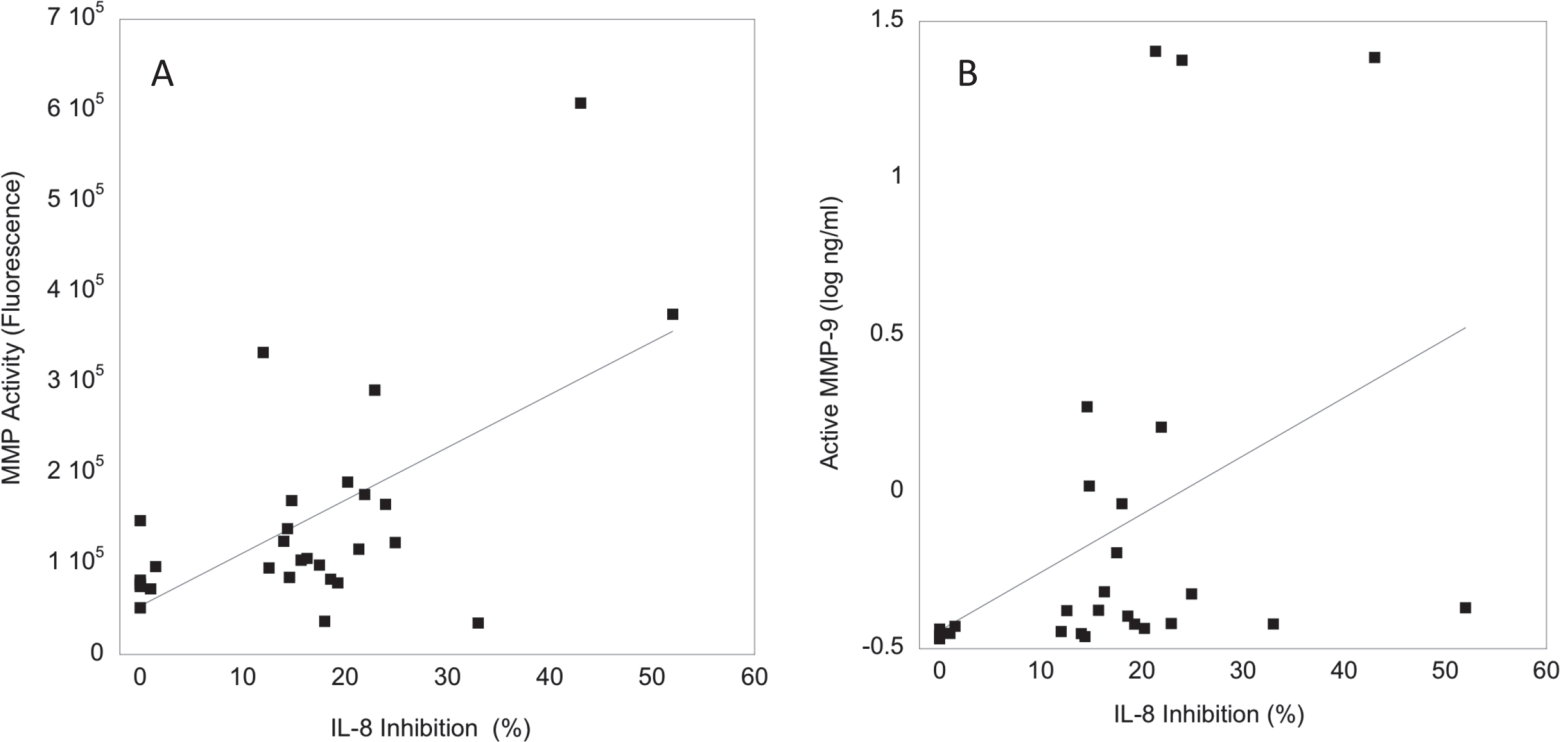
Relationship of active MMPs and IL-8 cleavage. A. The amount of MMP activity in genital fluids 22 samples positive for IL-8 cleavage and 5 negative for IL-8 cleavage was measured using a fluorogenic substrate and plotted against the % cleavage of IL-8 for each sample. B. The amount of active MMP-9 in the 27 samples was plotted against the % cleavage of IL-8 for each sample.

Since the total MMPs measured above contained both active and inactive MMPs, we analyzed active MMPs using a fluorogenic substrate that is cleaved by multiple MMPs. There was a significant correlation between IL-8 cleavage/alteration and the activity defined by the broad MMP substrate (Fig. 5A, Spearman r = 0.606, p = 0.02). Also, since MMP-9 has been previously associated with cleavage of IL-8, we compared IL-8 cleavage/alteration with active MMP-9. There was also a significant correlation between the activity and active MMP-9 levels (Fig. 5B, Spearman r = 0.690, p = 0.0001).

## Discussion

This study shows that an activity that can act on IL-8 is present in the lower genital tract fluid of some women. Inhibitor studies of samples collected from the two women with the highest activity strongly suggested that MMPs were causing IL-8 cleavage. Further, a strong positive correlation was observed between the IL-8 cleaving/altering activity and active MMP-9 levels as well as between the activity and cleavage of a substrate that can detect multiple active MMPs. Together these data, along with previous studies showing that MMP-9 can act on IL-8 [18], indicate that in most of the samples, MMPs and specifically MMP-9 was responsible for the IL-8 cleaving/altering activity. However, several of the samples that had relatively high levels of IL-8 cleavage/alteration had relatively low MMP-9 activity (Fig. 5B). Some of the samples with low MMP-9 activity had relatively high activity with the substrate that can be cleaved by multiple MMPs (Fig. 5A). Thus, our results suggest that in some of the samples, MMPs other than MMP-9 might participate in cleavage of IL-8. Several other MMPs have been reported to act on other chemokines [24]. For example, MMP-3 can cleave MCP-1 and MMP-8 can cleave CXCL5, although other MMPs have not been reported to act on IL-8 [24]. Alternatively, it is possible that other proteases, possibly either host- or bacterially-derived, could be present and act on IL-8 in the lower genital tract samples. A previous study of stability of IL-8 in lavage specimens showed that pooling lavages and incubating at 37°C for 24 h decreased IL-8 detection by about 50% [12]. However, in that study it is not clear that an enzymatic activity was responsible for the decreases in IL-8 and MMPs were not assessed.

To our knowledge this is the first report of active MMPs in the lower genital tract of women that shows the presence of multiple MMPs, although we did not test if all the MMPs were active. All of the tested MMPs including MMP-1, -3, -7, -8, -9, -10 and -12 were found in at least some of the women while none of the MMPs were found in all of the women. A study by Heng et al. [25] measured MMPs 1, -2, -3, -7, -8, -9, -12, and -13 and showed that MMP-7 was increased in labor, although MMP activity was not evaluated in that study. Active MMPs have multiple complex roles in inflammation including inhibition and potentiation [24], so that the active MMPs detected in our study could potentially alter responses to STDs or microbiota in the lower genital tract.

In this study, IL-8 cleaving/altering activity was observed in samples from both HIV-seropositive and –seronegative women. Thus, the presence of the activity does not appear to be associated with HIV-induced immunodeficiency. In fact, in the HIV-seropositive women, CD4 counts were higher in samples with activity than in samples with no activity. This could suggest that the expression of the protease in the genital tract is controlled through immune responses, and that the activity levels are consequently reduced in immunodeficient subjects. In contrast to our results, in a study of crevicular MMP-9 in HIV-infected subjects, a significant negative correlation between MMP-9 and CD4 levels was seen [26]. The IL-8 cleaving/altering activity was also not associated with types of bacterial microbiota. All but two of the nine positive samples had a microbiota that was dominated by *Lactobacillus*, which in previous studies has been associated with low levels of inflammation as determined by levels of pro-inflammatory cytokines [7,8,10,11,27–29]. However, it is possible that other infectious or non-infectious inflammatory stimuli could have induced the expression of the IL-8 cleaving activity in the women, but the women in this cohort were not tested for these. Neutrophils or macrophages could be the source of MMPs in our studies [24]. Interestingly, a cytolytic factor that can lyse neutrophils has been reported to be produced by *Gardnerella* [30]. Such a factor produced by genital bacteria could increase the levels of host-derived IL-8 degrading enzymes in the genital tract due to release from neutrophils.

Since IL-8 cleavage in many of the genital samples in this study was likely mediated by MMP-9, cleavage would be expected to substantially (approximately 10×) increase IL-8 biological activity as previously reported [18]. Due to the presence of endogenous IL-8 and other chemokines [7,28], and also potentially cleaved IL-8 in lower genital tract fluids, the bioactivity resulting from IL-8 cleavage could not be assessed in our study. A large increase in IL-8 bioactivity due to cleavage could substantially influence the amount of inflammation that occurs during STIs or other causes of lower genital tract inflammation that may have negative effects such as increasing HIV susceptibility [31]. Further study of the role that MMPs play in modulating genital inflammation are warranted, especially since inhibitors of MMPs are available that could be used to reduce pathogenic effects [32].
